# The family and substance abuser in the mental health institution environment

**DOI:** 10.4102/hsag.v29i0.2520

**Published:** 2024-10-08

**Authors:** Ruwayda Jacobs, Johanita Strumpher

**Affiliations:** 1 Department of Emergency Medicine, Faculty of Health Sciences, Nelson Mandela University, Gqeberha, South Africa; 2 Department of Nursing Science, Faculty of Health Sciences, Nelson Mandela University, Gqeberha, South Africa

**Keywords:** family member, psychiatric hospital, substance use disorder, rehabilitation, addiction

## Abstract

**Background:**

South African drug usage demographics are rare. Substance abuse disorder patients and their families have complex interactions. This study examines family members living with drug addict.

**Aim:**

The study seeks to investigate the experiences of family members living with a substance use disorder (SUD) individual.

**Setting:**

The research took place in a Gqeberha psychiatric facility. Low-income households were interviewed.

**Methods:**

This research is qualitative, exploratory, and descriptive study. Recorded and transcribed in-depth patient family interviews were collected. Qualitative thematic analysis was used to analyse data. The data themes were interpreted using family systems theory.

**Results:**

Each of the three main themes has subthemes. The first theme concerns family members’ inadequate comprehension of addiction. The subtheme explores family members’ denial and failure to recognize substance use disease symptoms. The second theme concerns variations in behaviour that challenge cultural norms. This subtheme addresses hallucinations, delusions, and fury in SUD patients. The third theme addresses the negative effects of drug use on families, potentially leading to divorce. This subtheme focuses on the family’s lack of awareness of resources that may have supported them and the SUD individual.

**Conclusion:**

Immediate family members are crucial to patient recovery. The family seemed to be struggling living with an SUD relative. Experts should help the family to manage the addict’s treatment. Healthcare education can help families cope with substance abusers.

**Contribution:**

This study will assist the nursing administration in discovering ways to help families manage a loved one with an SUD.

## Introduction

### Background of the study

Substance use persists in South Africa despite the National Drug Master Plan (2019–2024), the *Substance Use Disorder Act (2008)* and the *Mental Health Care Act (2002)*. Sustainable Development Goal (SDG) is the goal of the South African National Strategic Plan (NSP). The SDG aims to reduce non-communicable disease (NCD)-related premature deaths by 33% through prevention and treatment and improve well-being and mental health by 2030 to reduce premature disease, disability and death. It acknowledges the challenges of addressing the disease burden. All NCDs, including mental health, are addressed by the 5 × 5 approach in the Non-Communicable Disease Strategic Plan (NSP). Smoking, poor diet, inactivity, alcohol abuse and air pollution are also addressed in the National Strategic Plan (2022–2027).

The involvement of the patient’s family within the substance use disorder (SUD)’s therapy sessions can aid in long-term rehabilitation and avoid future relapses, according to Boyd and Luebbert (2020). The patient’s use of substances has a distinct and varied impact on each and every family and each of the family members. Some examples of this would include, but are not limited to, the unmet developmental needs of the patient or their family members (Lander, Howsare & Byrne 2013). Variables such as a damaged connection or relationship, economic difficulty, legal troubles, emotional pain and even violence being performed against the patient or their family members (Lander et al. 2013:8).

The health care system is inadequate, and the family is not recovering from addiction. Boyd and Luebbert (2020) recommended family involvement in SUD therapy to prevent relapse. Each family member suffers from unfulfilled developmental needs, disrupted relationships, economic difficulty, legal issues, emotional distress and even violence because of substance use (Lander et al. 2013:8). Ransom (2017) showed that poverty and parental negligence allow drug dealers to utilise the youth to sell drugs in South Africa. Traffickers exploit child drug users to sell more drugs. Gang leaders award fashionable clothes, shoes, food and anything they don’t have.

Family systems theory holds that the perspective of the family is seen as the primary relationship context in which individual character traits and ensuing patterns of behaviour are learned and reinforced. Family systems theory (FST) and the therapies derived from it view the symptoms of an individual as occurring within sequences of family interaction. An explicitly held conceptual precept that is accepted across family systems theoretical approaches is that there is a relationship between the identified patient’s symptom(s) and the total family interaction (Bowen 1978). Parents’ lives began to slip into a pattern of abnormal behaviour, according to a study done by Choate (2015). For example, parents, siblings and other members of the family might also begin to abuse substances. It might start with excessive drinking, behaving irresponsibly and self-medicating with over-the-counter prescription medication; it might manifest as insomnia, sleep apnoea or sleep disturbance, poor self-concept, lack of self-esteem, chronic fatigue and the list goes on and on. The family system as a whole was influenced by the drug addiction’s unpredictable behaviour. Their children’s behaviour became more unpredictable and dangerous, and the relationship and the bonds within the family (between siblings, the parent and the child) either collapsed or deteriorated to the point of no return. Parents felt increasingly helpless and vulnerable as these changes took place.

Choate also claimed that they would seek financial or emotional assistance from other family members, professionals or a combination of the two. Parents reported increasing desperation and an inability to cope successfully, but they discovered that seeking an expert’s advice was not always helpful. It added to the stress of coping. Systemic family theory and therapies pay close attention to repeating transactions that connect the problem behaviour of one person with the behaviour of other people within the family or other primary group (Ray 2018).

Substance use disorder individual would affect family dynamics, prompting outbursts and erratic behaviour. The SUD behaviour became unpredictable and harmful over time. The family relationships – between siblings, parents and the patient – collapsed or did not recover. These changes made parents feel helpless and insecure. Drug addiction’s unpredictable behaviour affected the family system. Their children’s behaviour became more unpredictable and riskier, and the family’s relationships (between siblings, parents and children) either dissolved or deteriorated (Bowen 1978). These changes made parents feel helpless and insecure.

Drug abuse can cause cognitive impairment, maladaptive personality and lasting brain damage. The family watches the SUD individual deteriorate and have damaged cognition. Addicts’ backgrounds might affect their emotional maturity, confidence, personal growth and life development. Peer pressure, physical and sexual abuse, early drug exposure, stress and parental guidance can also dramatically impact drug usage and addiction. Personality development, genetics and environment affect addiction and suicide risk at important developmental stages. Addicts often have mental, physical or emotional health issues. Bruijnen et al. (2018) studied 656 patients, 391 of whom used alcohol, 123 used cannabis, 100 used stimulants and 26 used opioids; 31% of patients were cognitively impaired. Alcohol users scored lower overall and in memory than cannabis users. Opioids impair visuospatial skills more than cannabis or stimulants. Younger patients performed better. No effect was found on the other qualities.

In a study conducted by Allan, Collings & Munro (2019), of the 67 people in the rehabilitation programme during the 12-month study period, 33% of participants were assessed and found to have cognitive impairments. Prior to their admission, all the participants completed a 7- to 14-day withdrawal programme period, and a medical practitioner oversaw the whole process to its completion. Participants with cognitive impairments were more likely to be younger and have left school by or before the age of 16 years old, compared to other participants without cognitive impairment (Allan et al. 2019). Retention rates were similar across both sets of non-impaired and impaired within the testing group (Allan et al. 2019). The participants with cognitive impairments were more likely to complete the programme than those without impairments; about 49% of participants did not complete the programme (Allan et al. 2019). However, this was not statistically significant, because of almost half of all the participants not completing the programme, and there was no difference in increased mental health for those with cognitive skills, who completed the programme.

### Problem statement

Families reported that they were unable to control the SUD individual’s behaviour. The individual’s behaviour was out of control and had a devastating effect on members of the family. The family was also unable to access help and support for themselves and the SUD individual. In a study conducted by Maina et al. (2021), the families reported that living with an individual who is an SUD patient tremendously impacts the caregiver’s emotional well-being, physical wellness and mental health. Because of the erratic and tremendously straining circumstances, the SUD individual would place the caregiver in.

The researchers noticed an increase in the number of individuals with SUD being admitted to the local acute psychiatric hospital presenting with psychiatric symptoms such as hallucinations, delusions and aggression. During the initial admission assessment, it was determined that they abused a variety of drugs which also included cannabis, mandrax, methamphetamine, heroin and cocaine. These individuals differed in age from 17 years to 39 years. Staff and clinical records reflect that these patients tended to relapse and were readmitted periodically.

## Research design and method

A qualitative, exploratory and descriptive study was undertaken to investigate the family’s experiences of living with a substance-abusing individual in the same home (Creswell & Creswell 2018). The qualitative researcher, according to Struwig and Stead (2017), is interested in ‘understanding the issues being researched from the view-points of the research participants’. This entails viewing the phenomenon under investigation via the participants’ eyes.

### Research question

These observations lead the researchers to ask the question: *‘What are the family experiences of living with substance use disorder (SUD) individual?*’

### Research objectives

The research objectives are given as follows:

- To explore and describe the family experience of living with a substance use disorder (SUD) individual.
- To make recommendations to the Department of Health regarding family support.

### Setting

Substance abuse has infiltrated many places within South Africa; however, certain areas do prevail with higher incidences of drug abuse cases (Minister of Police, Media Statements). The setting is the homes or areas of the relatives of the patients, who are living with an SUD individual in a lower sub-economic area in the Nelson Mandela Metropole.

### Study population and sampling strategy

The population was made up of family members, who shared a home with the individual who abused drugs like mandrax, methamphetamine and marijuana. The SUD individuals belonged to the categories of teenagers and adults. Participants were chosen through purposive sampling. There were mothers (10), dads (2), aunts (2), uncles (1), cousins (4) and sisters (1) amongst the participants, who had age ranges from 20 to 50 years old. Most of the participants were female family members and were from black and coloured racial background groupings.

### Data collection

The deputy manager of nursing, in the role of a gatekeeper, granted entry to the units where the names of potential participants were situated. Researcher 1 received the names of the likely family members from the nursing staff, which they obtained from the medical records. Researcher 1 assumed a training position at the hospital and had no prior knowledge or acquaintance with any of the patients or their family members. These families were invited for interviews, which were scheduled to be done on the hospital grounds a few days later. Prospective participants were informed about the study’s purpose as well as the data collection procedure. A written explanation was also provided to the participants.

After obtaining consent from the participants, the interviews were audio-recorded. Before the interviews, field notes were made that included the participants’ behaviour and what they said about the SUD individual. The interviews took place in a quiet and comfortable office within the hospital, where confidentiality was maintained. The interviews lasted between 60 min and 120 min. Data saturation was reached when no new themes or categories emerged from the interviews (Gray, Groves & Sutherland 2017).

Families were eager to speak with researchers because they felt compelled to convey their feelings. Semi-structured interviews were carried out. The researcher conducted interviews with individuals in both English and Afrikaans. She spoke both languages fluently. A general interview question asked was: ‘Tell me about your experience living with a family member who abuses substances’.

In addition to the question, ‘What assistance does your family require to manage the SUD individual?’. Despite the researcher’s intention to meet with only one family member, the majority of the interviews involved a minimum of two individuals. This is because family members feel the necessity to bring another family member along to provide assistance.

### Data analysis

After the interviews were conducted by researcher 2, researcher 1 transcribed the data collected. The data were then coded by both researchers, who used thematic analysis to find themes. According to Yanto (2023), thematic analysis refers to the focus on the search and generation of themes from the data set. The researchers compared their findings and achieved a consensus on the topics being explored.

Each transcript was examined using Tesch’s (1990) data analysis method. To gain a thorough understanding of the interviews, the researchers listened to audiotapes, and read and reread the verbatim transcripts multiple times. To create or rather conceptualise and validate related ideas from the transcripts, a consensus meeting was organised and held, whereby the themes that developed from the stories of the participants’ experiences were examined and backed up by relevant literature.

### Measures of trustworthiness

Trustworthiness is the degree of confidence in the honesty within the qualitative data provided and the certainty that the findings are based on factuality of the study methods, informants and context, which is referred to as credibility (Krefting 1991). In this study, four criteria were utilised to assure trustworthiness: credibility, transferability, conformability and dependability (Lincoln & Guba 1985), as follows:

- Credibility refers to the demonstration that the research study was conducted in such a manner that the research participants were accurately identified and described (Fouche, Strydom & Roestenburg 2021). Credibility was ensured by the extensive and varied experience of the authors. Researcher 1 works as an advanced psychiatric nurse in a mental facility, and researcher 2 is an academic who teaches undergraduate and postgraduate psychiatric nursing students. Reflexivity was used to avoid becoming unduly immersed in the experiences of the participants, which was upheld throughout the study. Member checking or peer checking was done with participants after the interviews had taken place to ensure that the final presentation of the data accurately reflects the experience. Peer reviewing and triangulation methods were used, which made use of several data sources, such as through interviews conducted and the keeping of reflective notebooks, doing pilot interviews, and ensuring a thorough descriptions of the approach and appropriate conclusions were made (Krefting 1991).
- Transferability refers to the extent to which the study’s findings can be applied to other studies and contexts (Fouche et al. 2021), which was ensured by providing full details of the study’s settings, data-gathering processes for participants, data collection time and triangulation (see credibility).
- Conformability implies that other researchers are able to confirm the origins of the derived findings and conclude that they would also come to the same or similar conclusions as this study (Fouche et al. 2021). Conformability was achieved through the concept of investigator triangulation by utilising the study team’s diverse ways (Krefting 1991). The researchers had built-up the research study’s confidence by providing a full description of the research approach and processes, allowing the study to be replicated in a variety of situations.
- The researchers, who had previously conducted qualitative research, coded the data using thematic analysis by finding themes. According to Gray et al. (2017), coding is a method of categorising and arranging information. As stated in Polit and Beck (2017), the researchers talked about their findings and came to an agreement on the main themes.
- Dependability, which can be described as evaluating through the examination of the evidence of the research process as found in the documentation, clearly formulated and published evidenced studies that are dependably used (Fouche et al. 2021). This was achieved through the use of a code and recode technique, which ensured dependability during the study’s analytical phase. After two weeks, the researcher returned to the data and re-analysed the information gathered. To improve the dependability, peer review was used.

### Ethical considerations

Ethical clearance to conduct this study was obtained from the Nelson Mandela University, Research Ethics Committee (Human) (No. H14-HEA-NUR-025) and the Department of Health granted permission to the researchers to conduct the study. Thereafter, the acting hospital manager granted permission for the research study to be conducted at the hospital. After being made aware of the objectives, benefits and limitations of the study, each participant’s individual consent was requested. All subjects provided written and informed consent before the study’s execution had taken place.

The ability to withdraw from the study at any moment and without consequence was made clear to the participants. None of the respondents declined to participate in the study. The participant’s privacy was protected by holding the family interviews within the hospital’s training school office. By not recording the participants’ names, anonymity was guaranteed. The obtained data were maintained in a password-protected folder with only the authors having access to it.

## Results

This section focuses on the three themes and subthemes that emerged (resulted) from the process of data analysis.

### Theme 1: The families were oblivious

Family members reported a routine and comfortable existence before their child was diagnosed with SUD. Family members typically described the SUD individual as quiet and reserved before acquiring the SUD. The developing SUD remained undetected by parents and family members. Only until it had caused significant behavioural changes in the individual, family members were neither prepared for nor equipped to deal with the occurrence.

#### Subtheme 1.1: Denial

Family members found it difficult to accept that their child might be developing or have an existing SUD, despite overwhelming evidence to the contrary. Initially, family members noticed that their child had acquired a new group of friends, started underperforming at school and that money and household items were frequently disappearing from the home without explanation. Challenging personal or family boundaries and breaking house rules were frequently explained away as normal teenager antics or acting out instead of seeing these behavioural markers as early indicators of an SUD individual:

’[*L*]ittle cheeky as they become at that age.’ (Transcription 2, mother)

To describe the increasing lack of respect for parental authority.

Declining academic performance and altered states of behaviour such as staying out later than usual or only coming back the following morning were attributed to influence from a new group of friends or other social factors:

‘The bad company’ (Transcription 6, sister)

‘[*B*]ut, now it is friends and then the peer pressure.’ (Transciption 1, mother)

[*S*]he was pole dancing and, in the morning, I saw her bringing money back home.’ (Transcription 1, mother)

The possibility of an SUD individual was not considered at first, as according to one parent:

‘[*I*]t was upsetting for us because we don’t expect him to be on drugs, he was raised and disciplined properly in a good home environment.’ (Transcription 6, mother)

Parents were either not educated enough to discern the warning signs or were in denial of the possibility of an SUD individual. Both denial and lack of education about SUD individual delayed the diagnosis of the affected individual and allowed the severity of the impact on the family unit to escalate over time.

### Theme 2: Behavioural changes in conflict with social norms

As behavioural changes escalated, parents began suspecting that these changes had other causes. According to one mother, her daughter cooked cannabis in a pot in the family home:

‘[*I*]t was cooked porridge. Inside, there were herbs and dagga [*cannabis*]. Then I asked my husband about it. This is dagga [*cannabis*], not herbs.’ (Transcription 3, mother)

‘[*W*]e took her to the drugstore and tested her; she tested positive for marijuana and methamphetamines. This is how we discovered it. Her personality has completely changed, and my child is like two different people.’ (Transcription 2, mother)

‘[*I*]t wasn’t until we forced him to get tested with the help of the police that we knew there was tik [*methamphetamine*], ecstasy, dagga, and heroin in his system.’ (Transcription 6, mother)

More significant symptoms appeared as the SUD individual progressed over time. Medically significant behavioural changes noted by the family increased in severity, exacerbating interpersonal, social and financial challenges within the family system. These included hallucinations, delusions and aggression (violent outbursts).

#### Subtheme 2.1: Hallucinations and delusions

Many family members mentioned perceptual abnormalities such as auditory and visual hallucinations. This is evident in the following quotes:

‘[*W*]hen he smoked the dagga, he heard the voices, he was running up and down like someone recharged his batteries. He also used to jump from one topic to the next.’ (Transcription 8, aunt)

Another participant mentioned that:

‘I don’t know why he says he is Mandela, that he is the president.’ (Transcription 4, mother)

Participants mentioned the delusions expressed by the individual with the SUD:

‘[*T*]he patient believes he has special abilities … he refers to us as the leaf people.’ (Transcription 10, father)

One family described the individual as having grandiose fantasies and frequently discussing believing in witchcraft:

‘[*H*]e enjoys discussing the aspect of being bewitched, witchcraft, and such topics.’ (Transcription 10, father)

Admission of the affected individual to mental health facilities became a predictable part of family life.

#### Subtheme 2.2: Aggressive behaviour displayed by the substance use disorder individual

Hallucinations and delusions coupled with the need for money to buy drugs led the individual experiencing the SUD to act aggressively towards their family members. The family’s refusal to give money to support the drug habit often precipitated aggressive behaviour. Twelve of the 13 participants described the SUD individual behaviour as abusive, violent and aggressive. According to one family, the individual planned to attack his aunt with an axe:

‘[*H*]e stated if I go home, I’ll take an axe and chop her into fine bits, as well as the aunty across the road.’ (Transcription 8, aunt)

Family members felt unsafe in their own homes because the SUD individual had unpredictable behaviour patterns:

‘[*H*]e’s already set fire to the house.’ (Transcription 5, father)

To safeguard the family from the affected SUD individual, one parent built a gate in the house that was locked at night:

‘[*I*] had to set up gates in my room so he wouldn’t come in.’ (Transcription 10, father)

There were many incidents where family members were assaulted:

‘[*H*]e threw me and shattered my ribs.’ (Transcription 5, father)

In one of the family interviews, there was a male cousin who used drugs and resided with his two female cousins along with their kids, in the same domicile. As a result of the cousin’s alleged threats to sexually assault or harm them, they claimed they were terrified of him.

### Theme 3: The abuser’s behaviour had a detrimental effect on the family

The families said that living with an SUD individual was a constant source of stress for them. The drug addicts exhibited erratic behaviours that the family did not or rather could not understand. Unacceptable behaviours ranged from headbanging, burning clothes and scaling the roof, amongst other examples of bizarre behaviours that induced terror and fear for the family members:

[*S*]he then sits on the kitchen cabinet like a child, her knees against her chest. She tosses the sugar into the container back and forth. She performs this around 3 o’clock in the evening.’ (Transcription 1, mother)

According to the families interviewed, these behavioural changes affected the entire family:

[*I*]f anything, the drug abuse does not affect only one member of the family. It [*referring to the drug abuse*] will have an impact on the entire family.’ (Transcription 6, mother)

Beyond the obvious health challenges faced by the affected individual, the situation resulted in a collateral decline in the physical, mental and emotional health of the other family members. Family ties became strained, and the social lives of family members were affected. Families faced judgement from their communities as evidenced by the comment made by one of the participants regarding her daughter:

‘[*Y*]ou carry the bible every day, but your house is not right.’ (Transcription 4, mother)

As stated by a community member to the mother about her child.

Families explained how their experiences with the individual affected by the SUD individual caused them to lose hope. Families indicated that before SUD appeared in their child’s life, family life had had its regular ups and downs. However, the abuse sustained and endured at the hands of the affected individual, coupled with the physical, emotional, financial, interpersonal and social strain leads to ineffective coping:

‘He didn’t sleep … I’m exhausted after not sleeping at night, and I have to go on duty again the next night. So now I’m not aware of what he’s doing, and he’s keeping his sister awake.’ (Transcription 6, mother)

Another parent mentioned that the abuse of substances made it impossible to live with his son:

‘[*I*]t, [*methamphetamine*] has released something within him, and we are finding it impossible to live with him.’ (Transcription 10, father)

#### Subtheme 3.1: Socioeconomic impact on the family

Some of the participants attempted to access help for the affected individual through drug recovery programmes. Individuals affected by SUD patients seldom complete the full recovery programme or fail to show significant long-term rehabilitation. These individuals often end up relapsing and end up being hospitalised, institutionalised or back in a rehabilitation facility later on. This can prove costly over time and put significant financial strain on a family facing many other challenges. One participant reported that she is losing all her savings and her home. The same family stated that their daughter’s behaviour had brought their marriage to the verge of divorce:

‘Yes … “our marriage was on the verge of disintegrating, and everything came apart”.’ (Transcription 2, mother & Transcription 2, father)

The SUD patient affected more than just the individual, and the ramifications lead to the disintegration of ties not only within the immediate family system but also the extended family and community.

In one case, the wife of one of the siblings refused to allow the SUD patient into their home:

[*H*]is wife did not want to open the door and then the abuser undressed and stood at the entrance of the complex.’ (Transcription 2, mother)

The community was not helpful with regard to the SUD patient. This is evident in the following quotes:

‘[*N*]ow, they ask questions at home, and they spread rumours and how all of a sudden now they are concerned. They did not have the decency to come to us and come and tell us. That is what I told them.’ (Transcription 6, mother)

## Discussion

Bowen (1978) studied the human family as a living organism. His goal was to match the theory’s ideals with living science. Chronic anxiety, basic life forces, emotional process and family as an emotional unit must be understood before understanding the theory’s eight aspects. The understanding of chronic anxiety, basic life forces, emotional processing and family as an emotional unit is imperative. The eight-part theory covers chronic anxiety, basic life forces, emotional process and family as an emotional unit (Bowen 1978). Chronic anxiety is caused by relational imbalances, and real or perceived risk causes anxiety in any person (Bowen 1978). Waini (2015) confirms that parental experiences and coping techniques with substance-abusing teenagers are scarcely recorded. Substance abuse is the excessive use of substances to the point where the person loses control of their behavior, although knowing it could damage their life. Which would explain why the family of the SUD individual remained uneasy throughout the SUD’s recovery and even after that as well.

Acute and persistent anxiety differs amongst family members because of potential threats causing short-term anxiety; however, threats can generate long-term anxiety if experienced over long periods of time (Syazrah et al. 2017). The family would react more to interpersonal disturbances than life events (Syazrah et al. 2017). Many SUD households have experienced chronic anxiety because of the family members’ fears of living with a drug user and their prior experiences with them (Syazrah et al. 2017). Psychotic patients who have had SUD for a long period of time and were not treated were prone to be hostile in nature (Syazrah et al. 2017). Choate (2015) remarked that the family’s peer relationships had changed, obscuring their child’s pre-drug behaviour.

Maina et al. (2021) suggested that addiction harms families; families of addicts’ struggle to stay together, while the SUD has destroyed countless families. Families invested time, effort and money to protect substance abusers and repair relationships. Drug use and parental stigma dominated the study; several psychological issues existed. The SUD child’s behaviour was impacted by the persistent and possibly lethal drug use.

The inconsistent essential lifeforces of the SUD patient’s family members hang in the balance while the SUD patient seeks out rehabilitation, because of the risky and violent behaviour of the SUD patient (Bowen 1978). In some cases, one family member shows constant disengagement and unprogressive qualities, while others show dedication, leaving it up to chance for what the SUD patient’s support structure would be like. Bowen (1978) saw humanity as diverse and united, sharing feelings, thoughts and bonds but also individuality wanting distinction or independence.

Deep emotional bonds make families more of emotional structures in nature. Bowen’s (1978) emotional family paradigm explores changes in long- and short-term emotional relationships, which unknowingly shape the family behaviour over generations. Family pathology predicts emotional instability as seen in the symptoms of emotional imbalances in the physical, emotional and social concerns of family members (Bowen 1978). Family emotional indicators include illness, unhappiness and crime, which lead to anxiety within the family (Bowen 1978).

Anxiety regulation of the family as a whole may have unanticipated side effects. However, no symptoms have been yet exposed within any research, because of the whole family not being able to release all their own anxieties of the experience at the same time (Boyd & Luebbert 2020). Symptom-based problem-solving removes societal disapproval and boosts creative impartiality, which is needed for trying to understand and relate to the SUD individuals and their circumstances leading to their SUD (Boyd & Luebbert 2020).

The eight interrelated components of Bowen Theory (1978) will be utilised to analyse the SUD individuals and their families:

1. Bowen’s (1978) theoretical self-differentiation scale assesses flexibility to overcome obstacles and attain goals. Self-distinction impacts relationship durability. This method helps vulnerable people change their thoughts, feelings and actions. The family must modify and inhibit their reactions to SUD individual’s unpredictable conditions, which can cause constant worry. Oversensitivity to others’ opinions, feelings and behaviours causes anxiety. As indicated by SUD individual-related family dynamics, unique people recover fast from acute worry, while less noticeable people can function stress-free (Bowen 1978). Each SUD individual’s situation requires family members to assess emotions and prepare answers. To preserve SUD individual’s rehabilitation, even if it affects family members mentally and emotionally (Bowen, 1978).
2. Triangles underpin human connections. Anxiety upsets a two-person relationship. Then, one or both dyad members often add a third person to relieve pressure. Three individuals provide anxiety with more outlets, and the initial connection feels better. When the three-person system can’t regulate worry, it forms interlocking triangles with others. Bowen researchers think biology uses triangles naturally. Triangles are good or harmful depending on how their members react to anxiety. Bowen thought that if one triangle member remained calm and emotionally connected to the other two, the system stabilises. However, stress and reaction lock members into a triangle and induce symptoms.
3. Nuclear family emotional process: Conflict, remoteness, over- and under-functioning reciprocity, and anxiety can produce spouse dysfunction and child focus. Parties dispute, blame and condemn. Distance between partners avoids emotional connection and uncomfortable themes. People aid each other in reciprocal connections. Two people are over- and under-adequate. Sickness or indecision can impair one partner. This theory discusses kid focus. Not being involved in one other’s personal lives helps people see things differently, but families don’t. Each family has the same experience, making it hard to see things differently (Bowen 1978). Because of their shared viewpoints and understanding, three-person systems cannot control worry and form interconnected triangles (Bowen 1978). As this three-way relationship cannot generate new solutions, Bowen (1978) thought triangulation is normal and can be helpful or detrimental depending on triangle partners’ anxiety management. According to Bowen (1978), one triangle member must be calm, another emotionally intelligent and the third rationally steady. Reactivity and stress might restrict and worsen symptoms and make triangle components resistant. Good results require the appropriate people and circumstances.
4. Family projection process: Nuclear families with worried parents have the fixed triangle. Parents try to adjust or hire a professional to ‘fix’ the child. Parents who manage anxiety and relationship concerns boost children’s functioning, say Bowen family systems experts. SUD individual received addiction therapy. How intellectual the family heads are will determine if difficulties can be resolved or if uncertainty will paralyse the family. Younger members focus on their seniors or parents, like in this study’s participants’ concern for their parents (Bowen 1978). SUD patients’ families often split over time because of positions taken on topics, which strains younger family members’ mental health (Bowen 1978).
5. Emotional cutoff: Highly distant relatives shun emotional contact. As emotional families separate, worry has less location to be absorbed in extended families, affecting future generations. This prolongs anxiety. People seek alternatives to disconnections. New ties increase symptoms. Some families cut links with the SUD individual because of irreparable damage. Syazrah et al. (2017) suggested that poor communication increases family-addict conflict, hindering rehabilitation. Under these conditions, families could not help SUD individual. According to this study, communication does not have an impact on the outcomes of addiction therapy for families. Despite their diligent attempts, the SUD individual did not get the intended communication. The SUD individuals experience a state of altered consciousness caused by drugs, which impairs their ability to engage in conversation or understand information (Bowen 1978). Family love and dedication make addicts feel valuable, so they seek recovery. The friction between addicts and their families breaks down communication.
6. Multigenerational transmission fosters self-differentiation. This text discusses multigenerational emotions. Beyond genetic and environmental factors, it considers family patterns. Triangle relationships reinforce family traits. Drug use was admitted by family. During the interviews, the father admitted that he was an alcoholic. Maintaining emotional distance helps families cope with life events (Bowen 1978). New relationships hurt SUD patients and their families. Over time, generational differences explain family feelings. When diagnosing SUD within the family dynamics, consider that heredity, environment and dynamics can vary over time (Bowen 1978). To prevent and help family members with SUD, family leaders must collaborate and educate. Families can share ideas and learn about history (Bowen 1978). This helps families understand and communicate about SUD patients.
7. The position of siblings influences the progression of fundamental and functional differentiation. These responsibilities are determined by the positions of their siblings, parents and other relatives; the eldest, youngest and middle children have distinct obligations within the family. In a family, the order of birth for siblings establishes the familial hierarchy. The concepts of marriage, adoption and biological relationship are commonly employed to define the term ‘family’. Engaging in reflective and independent thought can be quite challenging. Grief can manifest as a result of behavioural restrictions imposed by one’s family. When discord arises within a family, it tests the allegiance of its members. SUD individual assumed the roles of eldest, youngest and middle child, with some living with the family members who were their cousins, aunts and uncles.
8. Bowen concluded with societal emotion. Occasionally, social anxiety and instability peak. Overpopulation, resource shortages, epidemics, economic factors and a lack of survival skills can cause social regression. Bowen’s family systems theory found sociocultural emotions. Involuntarily, social anxiety and instability increased (Bowen 1978). Economic forces, epidemics, population increase, resource constraints and lack of survival skills can produce societal regression (Bowen 1978). In this study, the family believed long hospital stays would help them recover but neglected the social effects. Family members raised money for the mental health of their child. They underestimated funding delays leading to inconsistent rehabilitation, court fines, drug (hospital or pharmacy) costs, housing costs and unpaid family damages (McCann, Polacsek & Lubman 2019). Bowen (1978) found that social disadvantage had a negative impact on family interactions. Individuals suffering from SUD gradually lost their social standing. This limits their options for post-rehabilitation. Individuals with deviant motivations may repeat undesirable behaviour (Bowen 1978). Society has a greater impact on SUD individuals and their families than previously thought, making it difficult for SUD victims to succeed. The family stated that drug users have a negative impact on their mental health. This made it impossible to ignore SUD user in favour of therapy, putting pressure on families (Bowen 1978). SUD individual learned how to deal with high-stakes situations and modify family dynamics.

In this study, several families thought keeping the SUD individual in the hospital longer would help them recover through therapy. The family members had managed to acquire funds for the treatment of family members with severe mental illnesses. However, they did not realise the dangers of not being able to afford to sustain funding for longer periods of times than expected (McCann et al. 2019), leading to inconsistent rehabilitation, as well as court fines, drug (hospital or pharmacy) bills, housing expenses and family damages not being compensated (McCann et al. 2019). Being seen as poor in society had put major strain on the family’s standing within the communities they interacted with (Bowen 1978). The image of the patient with SUD suffered a significant decrease within society and continued to worsen with time. This does not augur well for their future endeavors, even if they successfully finish their rehabilitation. They may face ostracism and be viewed as social misfits due to their perceived malicious intents, which might ultimately result in their relapse (Bowen 1978). Societal implications play a far bigger role in the SUD patient and their family’s lives than expected, making it difficult for those affected by SUD to fully move-on and start new prosperous lives.

In addition, the family members spoke about the negative impact of living with someone who had an SUD, as well as the mental decline of the individual that they were living with., which made it difficult to cut-off or rather switch-off their feelings for the SUD patient and focus on solutions instead, thus causing great worry for family members (Bowen 1978). Overtime, the families of 13 SUD patients did see a considerable changes in their feelings as they grew more knowable and experienced in what to do in such high-stakes situations.

### Strengths and limitations

The nursing staff reviewed the medical records of persons diagnosed with SUD in order to identify appropriate candidates. The researchers reached out to the families by utilising the contact numbers obtained from the clinical records of the patients. Although the study primarily focused on methamphetamine, it was discovered that most people misused a variety of illegal substances. The research was conducted at a single government (public) hospital. The care patients received in government services differs from that received in private psychiatric treatment institutes. This can be seen as a limitation of the study as it focused only on one governmental (public) institution. This study gave us insight and understanding of the emotional pain and psychological triggers that families face and endured. What was terrible was the desperation that family members felt over their need for assistance for aiding their loved one and the fact that none is currently available at the treatment facilities in South Africa.

### Recommendations

**FIGURE 1:**
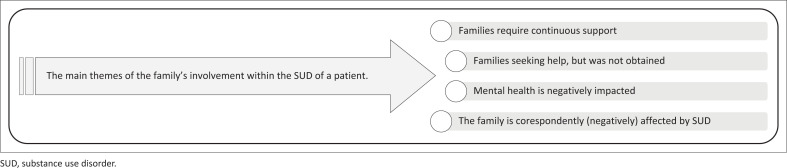
The main themes related to the family’s involvement with the substance use disorder individual.

Figure 1 describes the impact of SUD regarding the user’s family.

#### Community information systems

The families should be provided with information and resources on drug abuse and how to aid those who are addicted. The signs and symptoms of someone who is using illicit drugs need to be outlined and defined openly to the public. Social media can also be used as a platform to provide families with information regarding SUD. An inter-collaborative approach should be utilised in which primary health care nurses, police officials and social workers help and support parents whose children are abusing drugs. Facilities need to be made available to the public, whereby drug abusers can be referred to and where SUD individual can stay and be rehabilitated at.

In this study, parents need assistance to find outside-the-home accommodations for children with behavioural challenges. Each parent wanted to see their child recover in lengthy rehabilitation sessions. It also showed when parents reached their disorder-management limit and needed a remedy. According to Choate (2015), parents felt a change was needed after their prior attempts failed. The participants requested long-term care for the SUD individual. The family members had hoped that the SUD individual would improve if they were placed in a long-term facility. Awareness of the dangers of drug abuse and the high suicide risk that comes with it should be done through multiple public media platforms, such as through television, and social media, such as YouTube, Facebook, Twitter and Instagram. Public service announcements (PSAs) can be displayed through advertisements (such as billboards), social and print media (magazines and newspapers). Awareness campaigns can be run; workshops, school visitations at educational institutions, and church groups can facilitate awareness of acute and chronic danger of SUD individuals.

Effective counselling services should be made available to parents, caregivers and SUD individuals. Counselling is a tool that may be provided to the family members to provide them with skills to handle stressful situations. The family members in a study conducted by Marimuthu (2015) did not seek counselling or any other form of help to assist and support them in their plight. The family members experienced emotional challenges as well as felt helpless because of their situation. The parents benefited a lot from attending support groups as they improved their self-esteem, confidence and empowered them with knowledge. The support group also gave the parents reassurance about their common experiences, helping them to share ideas on how they can assist each other to cope with the circumstances of the SUD individual (Mathibela & Skhosana 2019).

In this study, family members typically reached out for formal help when they felt that the pressures arising from their child’s erratic behaviours were now far beyond their abilities and capacities to cope with. Most parents described the feeling of being powerless and that nothing they were trying seemed effective. When the barrier of not knowing was eliminated, reaching out for help made sense to parents and often they would speak about trying almost anything to find a solution for their child. Most family members tried to get some formal assistance for their youth through public health agencies or private resources (Choate 2015). Social workers in primary health care facilities should counsel the parents, as well as the SUD individual. Co-operation and compassion should be encouraged by social workers between families and the SUD individual to promote relationship building. Families require assistance from government services and resources at the grassroots (foundational) level (Ransom 2017).

Current parent support groups should be maintained. Parents should know about local support groups (Ransom 2017). Parents should establish and sustain a culture where families can communicate their issues (Ransom 2017). This lets family members learn from others. Parents of an SUD individual need support from policy or law. Parents should be advocated to be included in SUD individual and substance abuse laws. The government must protect parents from child and community violence as much as they must raise their children (Mathibela & Skhosana 2019). A hotline for family members to share their issues should also be created.

## Conclusion

This research study focuses on the family experiences of living with someone who abuses substances and has been hospitalised at a local psychiatric institution. Living with an addict hurts the family, according to the themes and subthemes outlined and explored in the study. The family experienced physical aggression, mental and social issues as a result of living with the SUD individual. To finance the patient’s drug habit, money and household belongings were stolen from the family members’ homes. Family members reported that they required counselling and help for themselves as well as the SUD individual but was difficult to obtain appropriate aid. Because the SUD’s time in an in-patient setting was limited, long-term rehabilitation was proposed as an option.

## References

[CIT0001] Allan, J., Collings, S. & Munro, A., 2019, ‘The process of change for people with cognitive impairment in a residential rehabilitation program for substance problems: A phenomenographical analysis’, *Substance Abuse Treat Prevention Policy* 14(1), 13. 10.1186/s13011-019-0200-y PMC6441186 30925888

[CIT0002] Boyd, M.A. & Luebbert, R., 2020, *Essentials of psychiatric nursing*, 2nd edn., Wolters Kluwer, Philadelphia, PA.

[CIT0003] Bowen, M., 1978, *The use of family systems theory in clinical practice. Comprehensive Psychiatry*, Jason Aronson, New York, NY. 10.1016/s0010-440x(66)80065-2 5922263

[CIT0004] Bruijnen, C.J.W.H., Dijkstra, B.A.G., Walvoort, S.J.W., Markus, W., Vander Nagel, J.E.L., Kessels, R.P.C. et al., 2019, ‘Prevalence of cognitive impairment in patients with substance use disorder’, *Drug and Alcohol Review* 38(4), 435–442. 10.1111/dar.12922 30916448 PMC6593747

[CIT0005] Choate, P.W., 2015, ‘Adolescent alcoholism and drug addiction: The experience of family members’, *Behavioural Sciences* 5(4), 461–447. 10.3390/bs5040461 PMC4695773 26529024

[CIT0006] Creswell, J.W. & Creswell, J.D., 2018, *Research design. Qualitative, quantitative, and mixed methods approach*, 5th edn., Sage, Thousand Oaks, CA.

[CIT0007] Department of Social Development, 2019, *National drug master plan (2019–2024) South Africa free of substance use disorder*, 4th edn., Government Printers, Pretoria.

[CIT0008] Fouche, C.B., Strydom, H. & Roestenburg, W.J.H., 2021, *Research at grassroots. For the social sciences human services professions*, 5th edn., Van Schaik Publishers, Pretoria.

[CIT0009] Gray, J.R., Grove, S.K. & Sutherland, S., 2017, *Burns & grove’s the practice of nursing research: Appraisal, synthesis, and generation of evidence*, 8th edn., Elsevier, St. Louis.

[CIT0010] Krefting, L., 1991, ‘Rigour in qualitative research: The assessment of trustworthiness’, *American Journal of Occupational Therapy* 45(3), 214–222. 10.5014/ajot.45.3.214 2031523

[CIT0011] Lincoln, Y.S. & Guba, E.G., 1985, *Naturalistic enquiry*, Sage, Beverly Hills, CA.

[CIT0012] Lander, L., Howsare, J. & Byrne, M., 2013, ‘The impact of substance use disorders on families and children: From theory to practice’, *Social Work in Public Health* 28, 194–205. 10.1080/19371918.2013.759005 23731414 PMC3725219

[CIT0013] Maina, G., Ogenchuk, M., Phaneuf, T. & Kwame, A., 2021, ‘“I can’t live like that”: The experience of caregiver stress of caring for a relative with substance use disorder’, *Substance Abuse Treatment, Prevention, and Policy* 16(1), 11. 10.1186/s13011-021-00344-3 33446208 PMC7809821

[CIT0014] Marimuthu, B.A., 2015, ‘An emotional rollercoaster. Vignettes of family members of illicit drug users’, *Acta Criminologica. Southern African Journal of Criminology* 3, 83–89.

[CIT0015] Mathibela, F. & Skhosana, R., 2019, ‘Challenges faced by family members raising adolescents abusing substances’, *Social Work/Maatskaplike Werk* 55(1), 87–107. 10.15270/55-1-697

[CIT0016] McCann, T., Polacsek, M. & Lubman, D.I., 2019, ‘Experiences of family members supporting a relative with substance use problems: A qualitative study’, *Scandinavian Journal of Caring Sciences. Empirical Studies* 33, 902–911. 10.1111/scs.12688 31033023

[CIT0017] Media statements, *Minister of Police*, viewed 28 January 2023, from www.gov.za/news/media-statements/police-minister-names-drug-hotspots-orders-police-get-tough-druglords-21-apr#:~:text=The%20Minister%20identified%20Chatsworth%20and,in%20dealing%20with%20druge20crime.

[CIT0018] Mental Health Care Act 17 of 2002, *Cape Town. Government Gazette*, 2002, vol. 449. No 24024.

[CIT0019] Polit, D.F. & Beck, C.T., 2017, Nursing research. Generating and assessing evidence for nursing practice, 10th edn., Wolters Kluwer, Philadelphia, PA.

[CIT0020] Struwig, F.W. & Stead, G.B., 2017, *Research: Planning, designing and reporting*, 2nd edn., Pearson South Africa (Pty) Ltd, Cape Town.

[CIT0021] Syazrah, F., Ghazalli, M., Ghani, N.A., Abdullah, B., Chik, W.M.Y.W., Ali, E.M.T.E. et al., 2017, ‘Patterns of interactions between family members and drug addicts’, *International Journal of Academic Research in Business and Social Sciences* 7(4), 303–312. 10.6007/IJARBSS/v7-i4/2807

[CIT0022] Ransom, S., 2017, ‘Illicit drug intervention at grassroots: A community upliftment model’, *Acta Criminologica: Southern African Journal of Criminology* 30(1), 65–79.

[CIT0023] Ray, W., 2018, *Family systems theory. The Wiley Blackwell Encyclopedia of family studies*, Wiley, New York, NY.

[CIT0024] Waini, A., 2015, *The challenges and coping resources of Family members whose children are addicted to chemical substances*, Master’s thesis, University of South Africa.

[CIT0025] Winters, K.C., Botzet, A., Dittel, C., Fahnhorst, T. & Nicholson, A., 2015, ‘Can Family members provide brief intervention services to their drug-abusing teenager?’, *Journal of Child & Adolescent Substance Use Disorder* 24(3), 134–141. 10.1080/1067828X.2013.777377 PMC4391737 25866459

[CIT0026] Yanto, E.S., 2023, ‘The what and how of essential thematic analysis’, *Qualitative Report* 28(11), 3120–3131. 10.46743/2160-3715/2023.6744

